# Couverture des cavités d´exentération: à propos de 20 cas

**DOI:** 10.11604/pamj.2022.43.105.26034

**Published:** 2022-10-26

**Authors:** Mahrouch El Mehdi, Oumkeltoum El Atiqi, Iman Yafi, Oumnia Ait Benlaassel, Smail Zinedine, Mehdi Geouatri, Mehdi Sahibi, Moulay Driss Amrani, Yassine Benchamkha

**Affiliations:** 1 Service de Chirurgie Réparatrice, Plastique et Brûlé, Centre Hospitalier Universitaire Mohammed VI, Marrakech, Maroc

**Keywords:** Reconstruction, lambeau, cavité d’exentération, tumeurs cutanées, Reconstruction, flap, exenteration cavity, skin tumours

## Abstract

La face est le support de la vie sociale. Le regard permet à l´individu d´interagir avec l´environnement et avec autrui. La cavité d´exentération résulte d´une chirurgie extrêmement délabrante qui laisse des séquelles esthétiques, fonctionnelles et psychologiques importantes. La couverture est obligatoire et doit répondre à plusieurs objectifs : combler la cavité et fermer les communications avec les régions voisines; obtenir une cicatrisation rapide; permettre une surveillance locale et enfin permettre au patient une réintégration sociale ainsi qu´une qualité de vie satisfaisante. Nous présentons notre expérience de comblement et de couverture de ces cavités. Nous avons pris en charge 20 patients présentant des cavités d´exentération sur une période de 5 ans (février 2015- février 2020) au sein de notre structure. Nous avons analysé les caractéristiques épidémiologiques, le profil clinique, les moyens de couverture et le devenir de ces patients dans notre structure. Notre moyenne d´âge est de 58,5 ans. Le carcinome épidermoïde est le type histologique prédominant suivi du carcinome basocellulaire justifiant l´exentération première. Les moyens de comblement des cavités étaient la cicatrisation dirigé dans 7 cas, le lambeau de fascia temporalis dans 2 cas, le lambeau de muscle temporal dans 10 cas. Les moyens de couverture étaient la cicatrisation dirigée dans 6 cas, une greffe de peau totale dans un cas, le lambeau médio-frontal dans 6 cas (seul ou associé au muscle temporal), un lambeau scalpant de converse dans trois cas. Un lambeau de grand dorsal libre était utilisé dans deux cas. Treize patients ont présenté une bonne évolution avec un recul moyen de 9 mois avec une bonne cicatrisation. La complication la plus fréquente est l´infection et le lâchage de suture. Deux patients sont perdus de vue. Il existe plusieurs moyens de reconstruction de ces cavités. Le lambeau de muscle temporal apporte robustesse et sécurité et constitue une excellente solution de comblement selon notre expérience. Les prothèses sont une solution esthétique mais coûteuse qu´il faudra développer dans notre contexte.

## Abstract (en)

The face is social support. Eyes allow the individual to interact with the environment and others. Orbital exenteration defects are the results of extreme disfiguring surgery, leading to functional, aesthetic and psychological sequelae. Reconstruction is essential and must meet several objectives: filling the cavity and closing any communications with the adjacent structures, obtaining rapid healing, allowing for local monitoring and finally enabling patients to reintegrate into society and achieve a satisfactory quality of life. We here present our experience in filling and reconstructing these cavities. Our study involved 20 patients with orbital exenteration defects over a period of 5 years (February 2015-February 2020). We analyzed the epidemiological features, clinical profile, and methods of the reconstruction as well as patients´ outcomes in our hospital. The average age of patients was 58.5 years. Squamous cell carcinoma was the main histological type, followed by basal cell carcinoma. Filling techniques included directed wound healing (7 cases), temporalis fascia flap (2 cases), temporalis muscle flap (10 cases). Methods of reconstruction included directed wound healing (6 cases), full-thickness skin graft (1 case), mediofrontal flap (6 cases; alone or associated with the temporalis muscle), converse scalping flap in three cases. Free latissimus dorsi flap was used in two cases. Thirteen patients showed good outcomes, with good healing after an average follow-up period of 9 months. The most common complication was infection and suture release. Two patients were lost to follow-up. Orbital exenteration defects can be approached in many ways. Temporalis muscle flap has been shown to be a robust and safe choice as well as an excellent filling solution. Prostheses are an aesthetic but expensive solution that should be developed in our hospital.

## Introduction

La face joue un rôle social et fonctionnel capital. L´exentération orbitaire se définit comme l´ablation du globe oculaire et de ses annexes (paupières, muscle, graisses) [1]. Elle peut être simple ou dite élargie emportant en plus du globe et de ses annexes, l´os et les structures voisines. C´est une chirurgie délabrante indiquée le plus souvent dans la prise en charge des tumeurs naissant dans/ou envahissant la cavité orbitaire. Les pertes de substances résultantes sont souvent complexes, mettant en jeu des structures adjacentes de la face. La couverture de ces cavités d´exentération se fait le plus souvent en deux temps après contrôle histologique des marges d´exérèse. C´est un véritable challenge qui a pour objectif de combler la cavité et de fermer les communications avec les régions voisines en protégeant le massif osseux orbitaire; d´obtenir une cicatrisation rapide capable de résister à une éventuelle radiothérapie; de permettre une surveillance locale afin de détecter d´éventuelles récidives et enfin permettre au patient une réintégration sociale ainsi qu´une qualité de vie satisfaisante. L´objectif esthétique est aussi certain et doit permettre de redonner à la face un aspect le plus harmonieux possible. Le comblement de la cavité fait appel à plusieurs techniques chirurgicales en chirurgie plastique, du plus simple au plus compliqué, allant de la cicatrisation dirigée aux lambeau musculaire et musculo-cutané régionaux, au lambeau libre à distance [1,2]. Nous rapportons ici notre expérience concernant le comblement et la couverture de ces cavités d´exentération au sein de notre structure.

## Méthodes

Il s´agit d´une étude rétrospective sur une durée de 5 ans (février 2015- février 2020) concernant 20 patients opérés au sein du service de chirurgie réparatrice et plastique du CHU Mohammed VI de Marrakech. Le consentement éclairé de chaque patient a été obtenu pour la confection et l´exploitation du dossier médical à but scientifique. Les critères d´inclusion étaient les patients présentant une cavité d´exentération secondaire à une exérèse de tumeurs cutanées malignes ; les patients ayant bénéficié d´exentération dans notre structure et ailleurs ; les cavités d´exentération avec ou sans exposition de structures nobles. Les données sont recueillies et analysées à l´aide du logiciel Microsoft Office Excel.

## Résultats

**Profil épidémiologique:** au total, 20 patients ont été pris en charge dans notre structure. Seize hommes et 4 femmes pour un sexe ratio homme/femme de 4. La moyenne d´âge est de 58,5 ans avec des extrêmes allant de 27 à 84 ans. Quatre-vingt-deux pourcent (82%) de nos patients proviennent d´une zone rurale contre 18% d´origine urbaine. Deux de nos patients, âgé de 28 et 11 ans, présentaient un terrain de *Xeroderma pigmentosum*.

**Profil histologique et radio-clinique:** le type histologique prédominant était le carcinome épidermoïde dans 36% des cas, le carcinome basocellulaire dans 27%, le méningiome dans 5,8% et le carcinome mucineux dans 5,8% des cas (Tableau 1). La tomodensitométrie (TDM) orbito-faciale était systématique, permettant d´explorer l´envahissement des muscles orbitaires, de la graisse extraconique et/ou intraorbitaire, du globe oculaire, de l´os afin de justifier l´exentération. Pour les carcinomes spinocellulaires, le bilan d´extension était complété par un scanner orbito-facial et thoraco-abdomino-pelvien. Le type de cavité d´exentération prédominant dans notre série sont les cavités de type III et IV selon Kesting *et al*. exposant des structure osseuses voisines et/ou brèches sinusiennes. Les pertes de substances exposaient des structures sous-jacentes chez 10 (50%) de nos patients. Les structures exposées étaient le cadre orbitaire chez 8 patients, le sinus frontal dans 2 cas, et le sinus maxillaire dans 2 cas.

**Tableau 1:** profil thérapeutique de nos patients

Cas	Sexe	Age	Type histologique	Exposition de structures	Délai de couverture	Geste de couverture	Complications	Suivi post opératoire
13	F	68	CBC	Cadre orbitaire	Différer	Lambeau muscle temporal/ médio-frontal	----	Bonne cicatrisation a 6 mois
14	M	27	Méningiome grade 1	Non	Différer	Lambeau de muscle temporal/ lambeau médio-frontal	----	Bonne cicatrisation à 6 mois
15	M	73	CBC	Sinus frontal	Différer	Lambeau de muscle temporal / lambeau Mustardé + greffe cutanée	----	Bonne cicatrisation à 6 mois
16	M	42	Carcinome épidermoïde	Cadre orbitaire+ brèche sinus maxillaire	Différer	Lambeau muscle temporal/ médio-frontal	----	Perdu de vu
17	M	70	CBC	Cadre orbitaire + sinus frontal	Immédiate	Lambeau de muscle temporal / scalpant de converse	----	Bonne cicatrisation à 4 mois
18	M	61	Carcinome épidermoïde	Non	Différer	Lambeau de muscle temporal/ lambeau scalpant de converse	----	Radiothérapie adjuvante
19	M	58	CBC ulcéré	Paroi orbitaire interne	Différer	Lambeau grand dorsal libre	Nécrose du lambeau	Cicatrisation dirigée + greffe cutanée secondaire
20	M	48	CBC	Oui	Différer	Lambeau grand dorsal libre	----	Bonne cicatrisation

**Figure 1:**
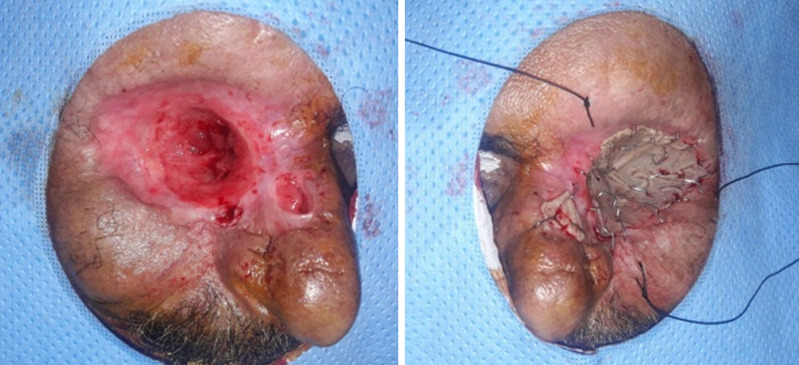
cicatrisation dirigée et greffe de peau totale (cas n°1)

**Figure 2:**
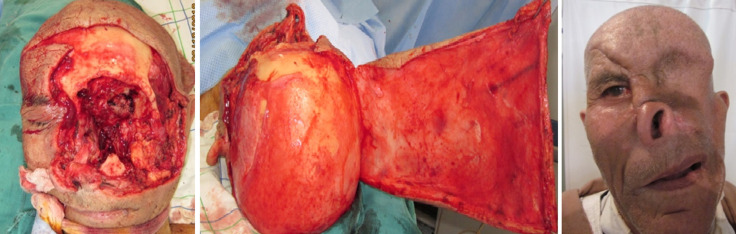
cavité de type IV couverte par muscle temporal + lambeau scalpant de converse (résultat à 2 ans (cas n°17)

**Profil thérapeutique:** quatorze de nos patients ont bénéficié d´une exentération dans notre structure tandis que les 6 autres nous ont été adressés (service d´otorhinolaryngologie) pour couverture de leur cavité. Chez la majorité de nos patients (19 cas) la couverture était différée après contrôle histologique des marges d´exérèse, tandis que pour un patient la couverture était immédiate après exentération. Les moyens de comblement des cavités étaient la cicatrisation dirigée dans 5 cas, le lambeau de fascia temporalis dans 2 cas, le lambeau de muscle temporal dans 10 cas. Les moyens de couverture étaient la cicatrisation dirigée dans 6 cas, une greffe de peau totale dans un cas (Figure 1), le lambeau médio-frontal dans 6 cas (seul ou associé au muscle temporal), un lambeau scalpant de converse dans trois cas (Figure 2). Un lambeau de grand dorsal libre était utilisé dans deux cas permettant à la fois le comblement et la couverture.

**Figure 3:**
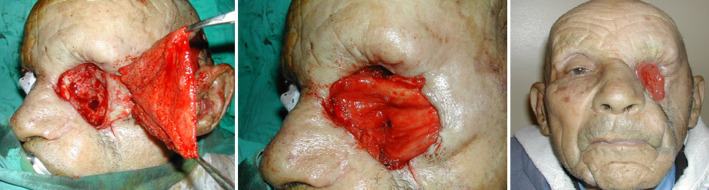
comblement par lambeau de fascia temporalis et couverture par cicatrisation dirigée (résultat à 4 mois)

**Figure 4:**
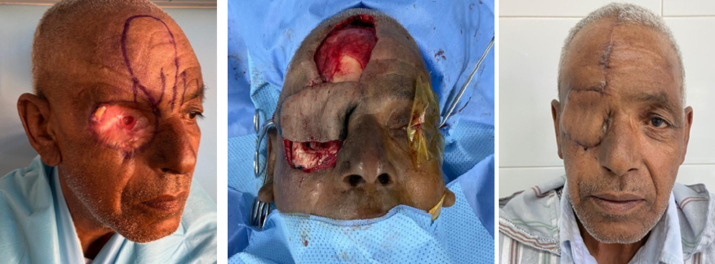
couverture par lambeau médio-frontal (résultat à 4 mois (cas n°2))

**Figure 5:**
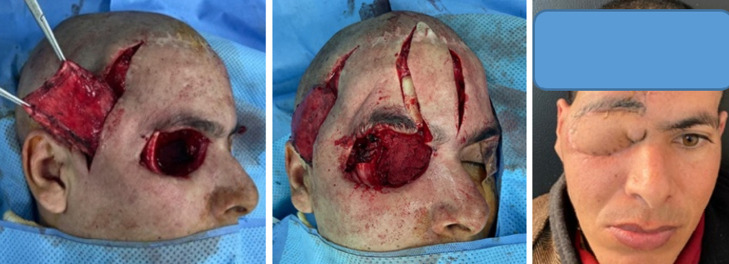
lambeau de muscle temporal associé à un médio-frontal

**Technique chirurgicale:** la cicatrisation dirigée est menée par des pansements pro-inflammatoires jusqu'à fermeture de la cavité. Elle est utilisée pour couverture dans cinq cas et pour préparer la cavité à un geste ultérieur chez 5 patients. La greffe de peau totale était réalisée sur un bourgeon homogène et saignant, prélevé à la face interne du bras chez un de nos patients. Le lambeau médio-frontal était utilisé après avoir repéré à la sonde Doppler des axes vasculaires supra-trochléaire et supraorbitaire de part et d´autre, puis mesure et dessin du patron de la PDS. Le lambeau est transposé sur la PDS après rotation de 180° et fixé en 2 plans. Le lambeau de fascia temporalis est abordé après repère de l´artère temporale superficielle au Doppler puis il est transféré sur la cavité par-dessus le cadre orbitaire externe chez les deux patients (Figure 3). L´utilisation du muscle temporal était faite selon la technique habituelle par une incision hémi-coronale, lever du lambeau musculaire puis transfert sur la PDS orbitaire après ostéotomie de la paroi latérale de l´orbite ou par-dessus (Figure 4, Figure 5). Le grand dorsal était utilisé en lambeau libre chez deux de nos patients pour des pertes de substances hémifaciales droite et gauche secondaires à l´exérèse large de la tumeur initiale.

**Figure 6:**
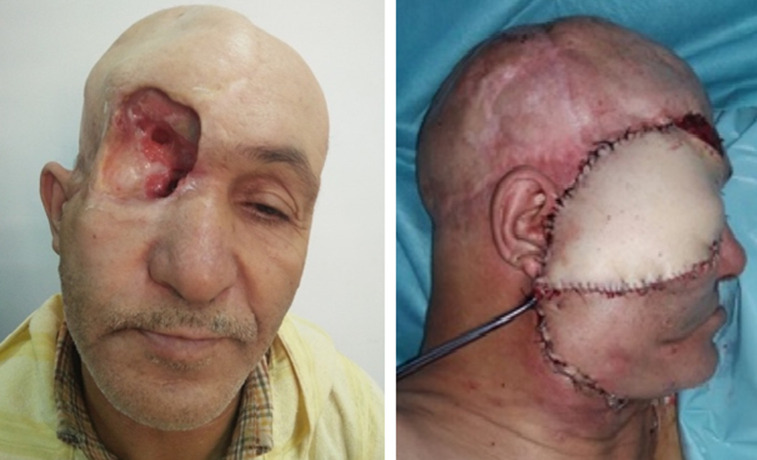
reconstruction par lambeau de grand dorsal libre (cas n°19)

**Évolution:** trois patients ont eu une radiothérapie adjuvante. Avec un recul moyen de 11 mois, la couverture des cavités d´exentération était de bonne qualité avec une bonne cicatrisation pour 76,5% de nos patients. Deux patients ont été perdus de vue. Aucun de nos malades n´a bénéficié d´appareillage par épithèse. Les complications de la couverture étaient marquées par deux infections locales et lâchage des berges, traitées par des soins locaux et une antibiothérapie adaptée, et une nécrose de lambeau de grand dorsal libre traitée par parage et cicatrisation dirigée (Figure 6).

## Discussion

L´exentération est une chirurgie délabrante à l´origine d´une grande déformation orbitaire. Elle peut se compliquer d´une fistule orbitosinusienne et/ou orbitonasale, d´une exposition ou d´une brèche dure mérienne pouvant se compliquer d´infections méningées mettant en jeu le pronostic vital. Kesting *et al*. [1] proposent une classification des cavités d´exentération selon l´exposition ou non des structures voisines. Les type I sont les cavités secondaires à une exentération simple, les types II sont les cavités avec pertes et/ou expositions du cadre orbitaire, les types III et IV sont les PDS exposant des structures osseuses avoisinantes ou avec brèche sinusienne. Dans notre série, ce sont les cavités de type III et IV qui prédominent. L´objectif de la reconstruction est avant tout fonctionnel, afin de séparer et de protéger les structures sous-jacentes, puis elle doit être aussi esthétique que possible. Le choix de la technique chirurgicale va dépendre aussi bien du type de cavité, que du patient et de son terrain.

La cicatrisation dirigée n´est pas utilisée dans la série de Benazzou S *et al*. [2] en raison de cavité exposant des structures sous-jacentes. Selon Kesting *et al*. elle doit être réservée aux cavités de type I chez des patients pouvant avoir un terrain fragilisant la peau. Dans notre série, elle a été utilisée pour deux cavités chez un patient *Xeroderma pigmentosum*. La greffe cutanée est très peu utilisée dans la couverture de ces défects dans notre série, tout comme la série de Benazzou S *et al*. car 53% de leurs tumeurs étaient classées T4 conduisant après exérèse à une exposition dure mérienne et/ou sinusienne. Ainsi, selon Ameya A *et al*., la greffe de peau totale doit être réservée aux cavités d´exentération de type I, chez un patient qui ne sera pas irradié en post-opératoire [3]. Le lambeau médio-frontal possède une bonne vascularisation par l´artère supra trochléaire et supraorbitaire. Il atteint facilement la cavité et constitue une bonne option thérapeutique assurant à la fois fonction et aspect cosmétique satisfaisant [4]. Il a été utilisé chez 2 de nos patients en seule option de couverture avec succès et bonne cicatrisation, et associé à un muscle temporal dans 3 cas.

Le lambeau de fascia temporalis est un lambeau situé juste sous le plan cutané et basé sur l´artère temporal superficiel. Il apporte un sous-sol rigide, vascularisé et permet de recevoir facilement une greffe cutanée [3,5,6]. Le lambeau de muscle temporal est une technique simple, qui apporte robustesse du sous-sol et sécurité vasculaire [7]. Pour Ameya *et al*., il peut être utilisé avec grande sécurité pour les défects de type III et IV. Sur une série de 15 patients de la série de Benazzou S *et al*., dix ont bénéficié d´un lambeau temporal musculaire faisant de ce lambeau la technique de choix. Pour nous, ce procédé a été utilisé chez 10 patients qui présentaient des cavités de type III avec des suites post opératoires simples et une bonne évolution locale. Il comble aisément la cavité d´exentération et autorise une radiothérapie précoce [8]. Le lambeau de grand dorsal libre permet de reconstruire les grandes PDS de l´hémiface et apporte une sécurité vasculaire en raison du grand calibre de son pédicule [2,9,10]. Deux patients ont bénéficié de ce lambeau libre avec un cas de nécrose post opératoire. Il n´a été utilisé qu´à deux reprises en raison du plateau technique insuffisant dans notre contexte et du terrain des patients contre-indiquant une chirurgie longue. Concernant les épithèses, les patients préfèrent souvent le port d´un pansement oculaire. Selon Benazzou *et al*. seulement 47% des patients auxquels on avait confectionné des prothèses les portaient régulièrement. Dans notre contexte, le manque de plateau technique et le niveau socio-économique des patients font de l´épithèse une option difficile à réaliser. Cependant elle constitue une solution simple et fiable, qui permet d´éviter une chirurgie lourde à des patients âgés, et qui donne d´excellents résultats esthétiques [11]. La limitation de notre étude pourrait se trouver dans le contexte socio-économique et le manque d´accès aux soins dans notre contexte qui pourrait influencer le fait que la majorité de nos patients étaient satisfaits des résultats à long terme, bien qu´aucune épithèse oculaire n´ait été réalisée.

## Conclusion

Les lambeaux locorégionaux restent une option fiable qui donnent de bon résultats fonctionnels et esthétiques. La greffe cutanée est une bonne solution pour les patients aux cavités de type I et non destinés à être irradiés. Pour des patients ayant une exposition osseuse ou sinusienne, le lambeau de muscle temporal apporte robustesse et sécurité, et constitue une excellente solution de couverture qui résiste à l´irradiation adjuvante. Enfin, les prothèses sont une solution simple et qui donne un résultat esthétique satisfaisant.

### Etat des connaissances sur le sujet

- De nombreuses techniques chirurgicales permettent la réhabilitation de la cavité orbitaire;
- Le choix de chaque technique dépend du type de cavité d´exentération.

### Contribution de notre étude à la connaissance

- On souligne la robustesse du lambeau musculaire temporal;
- La satisfaction à long terme des patients ayant été traités par lambeaux locorégionaux.

## Remerciements

Nous remercions à travers cette étude tous les membres de l´équipe du service de chirurgie réparatrice et plastique du CHU Mohammed VI de Marrakech, dirigé par notre cher Professeur Benchamkha, qui a permis que ce travail soit mené dans un cadre à la fois rigoureux et éthique vis à vis des patients.
